# Calcitonin Gene-Related Peptide Modulates Heat Nociception in the Human Brain - An fMRI Study in Healthy Volunteers

**DOI:** 10.1371/journal.pone.0150334

**Published:** 2016-03-18

**Authors:** Mohammad Sohail Asghar, Lino Becerra, Henrik B. W. Larsson, David Borsook, Messoud Ashina

**Affiliations:** 1 Danish Headache Center and Department of Neurology, Glostrup Hospital, Faculty of Health and Medical Sciences, University of Copenhagen, DK-2600, Glostrup, Denmark; 2 P.A.I.N. Group, Boston Children’s Hospital and Center for Pain and the Brain, Harvard Medical School, Boston, Massachusetts, 02453, United States of America; 3 Functional Imaging Unit, Department of Diagnostic, Glostrup Hospital, Faculty of Health and Medical Sciences, University of Copenhagen, DK-2600, Glostrup, Denmark; University of North Dakota, UNITED STATES

## Abstract

**Background:**

Intravenous infusion of calcitonin-gene-related-peptide (CGRP) provokes headache and migraine in humans. Mechanisms underlying CGRP-induced headache are not fully clarified and it is unknown to what extent CGRP modulates nociceptive processing in the brain. To elucidate this we recorded blood-oxygenation-level-dependent (BOLD) signals in the brain by functional MRI after infusion of CGRP in a double-blind placebo-controlled crossover study of 27 healthy volunteers. BOLD-signals were recorded in response to noxious heat stimuli in the V1-area of the trigeminal nerve. In addition, we measured BOLD-signals after injection of sumatriptan (5-HT_1B/1D_ antagonist).

**Results:**

Brain activation to noxious heat stimuli following CGRP infusion compared to baseline resulted in increased BOLD-signal in insula and brainstem, and decreased BOLD-signal in the caudate nuclei, thalamus and cingulate cortex. Sumatriptan injection reversed these changes.

**Conclusion:**

The changes in BOLD-signals in the brain after CGRP infusion suggests that systemic CGRP modulates nociceptive transmission in the trigeminal pain pathways in response to noxious heat stimuli.

## Introduction

Calcitonin-gene-related-peptide (CGRP) belongs to a family of peptides including adrenomedullin, amylin and calcitonin with diverse biological functions in the peripheral and in the central nervous system [1, 2] Immunohistochemial studies have demonstrated that CGRP is present in the trigeminal ganglion and trigeminal nucleus caudalis [3–5] CGRP is also released into the extracerebral circulation of humans during thermocoagulation of the trigeminal ganglion [6]. Furthermore, CGRP is a potent vasodilator of human arteries [7] and mediates relaxation of these arteries via activation of the CGRP(1)-type receptor [8]. A dense supply of CGRP-containing fibers is present around cerebral vessels and is believed to originate in the trigeminal ganglion [9]. The role of CGRP in neurovascular headache has been extensively studied in human provocation experiments [10–12]. Intravenous infusion of CGRP causes headache [10, 13] and dilatation of extracranial arteries [10] in healthy volunteers and migraine-like attacks in patients with migraine [11, 14, 15]. To what extent intravenous CGRP modulates nociceptive processing in the brain is unknown.

Blood-oxygenation-level-dependent (BOLD) functional magnetic resonance imaging (fMRI) is widely used to hemodynamically map responses to pain in humans [16, 17]. A recent fMRI study showed that CGRP and sumatriptan did not modulate visual processing in humans and neither systemic CGRP nor sumatriptan has an effect on the BOLD-signal *per se* [18]. The latter is an important observation because CGRP is a strong vasodilator while sumatriptan is a strong vasoconstrictor and thereby theoretically may have an intrinsic effect the hemodynamic dependent BOLD-signal [10].

To date, no studies have investigated changes in the BOLD-signal in pain related brain-regions after CGRP infusion. In the present study, we hypothesized that intravenous infusion of CGRP would cause changes in the modulation of pain processing in the brain in response to noxious heat stimuli. In addition, we hypothesized that sumatriptan, a 5HT_1B/1D_ antagonist, would reverse these changes. To test this we conducted a double-blind placebo-controlled randomized crossover BOLD-fMRI study in healthy volunteers. We applied noxious heat stimuli to the trigeminal area of V1 and recorded BOLD-signals in the brain before and after drug administration.

## Material and Design

### Volunteers

Thirty-one healthy volunteers were recruited to the study. We recruited participants via an announcement on a Danish website for recruitment of volunteers to health research *(*www.forsoegsperson.dk*)* and though a magazine for university students *(MedicinerOrganisationernes Kommunikationsorgan (MOK))*. Healthy volunteers between ages 18–55 years were eligible for inclusion in the study. Exclusion criteria were: history of a medical disorder; migraine or any other type of headache (except episodic tension-type headache less than once a month); daily intake of any medication except contraceptive; pregnant or nursing women, contraindications for MRI scans (e.g., metal fragments, claustrophobia), cardiovascular or cerebrovascular disease, or uncontrolled psychiatric disease or drug misuse. Heavy smokers and caffeine users were likewise not included in the study. All female participants used safe contraceptive methods. Enrolment was performed at Glostrup Hospital, Glostrup, University of Copenhagen; Denmark.

The Ethical Committee of Copenhagen (H-KA-20060083) approved the study. All participants gave written consent after receiving detailed oral and written information and the study was conducted in accordance with the Helsinki II Declaration of 1964, as reversed in Edinburgh in 2000. The study was in addition registered in clinicaltrails.gov (NCT00363532).

### Pharmacological Interventions

All participants were investigated twice and received infusion of 1.5 μg/min h-αCGRP (Calbiochem–Merck4Biosciences) at one investigation day and placebo (isotonic saline) at the other investigation day, the order being randomized. Simple randomization was applied. The medicine (active and placebo) was delivered in sequentially numbered containers for each study day. The randomization code was kept separate and remained sealed until end of the trail. The randomization and packing was preformed by the hospital pharmacy. Infusion took 20 min. Infusion of 1.5 μ**g/min of** CGRP over 20 min reaches its maximum vascular effects after 30 to 45 min. Vascular changes can be measured already after 15 min and lasts between 90–180 min[12]. The two investigation days were separated by at least one week. On both experimental days the participants received 6 mg subcutaneous injection of sumatriptan (Imigrain® injection, GlaxoWellcome Operations, Bernard Castle, UK) 40 min after start of h-αCGRP or placebo infusion.

### Experimental procedures

All participants reporting to the laboratory were headache free. Coffee, tea, cocoa or other methylxanthine-containing foods, beverages, and tobacco was not allowed for at least 12 h before start of the study. Subjects were placed in the supine position in the MRI room and a venous catheter (Venflon®) was inserted in to the left antecubital vein for infusion. We collected blood samples to determine the baseline hematocrit, potassium and sodium levels. The subjects were monitored with ECG, end-tidal CO_2_ (capnograph, Datex, Finland), blood oxygen saturation, blood pressure and heart rate (Veris monitor, Medrad, USA). MR imaging was performed on a 3.0 Tesla Philips Achieva Scanner (Philips Medical Systems, Best, The Netherlands) using a one-element phased-array receive head coil. We first obtained a reference anatomical whole-brain image and then repeatedly measured the BOLD-signal after noxious heat stimulation.

**Fig 1:**
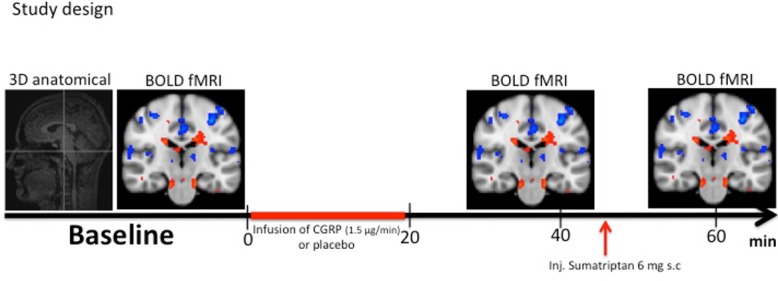
Study design: All variables were recorded at fixed time points throughout the study. The anatomical image was recorded at T_-15_. BOLD-fMRI scan after noxious heat stimulation with the thermode was recorded at T_-5_ (baseline), at T_40_ and T_60_ (15 min after sumatriptan injection). Start of infusion was defined at T_0_. According to the randomization code CGRP (1.5 μg/min) or placebo was infused over 20 min. Intervention with subcutaneous injection of sumatriptan was performed at T_45_._._Hemodynamic variables, adverse event and headache intensity on the verbal rating scale (VRS) was recorded at 0, 5, 10, 15, 25, 35, 45, 55 and 65 min.

We defined time of drug administration as T_0_. All variables were recorded at fixed time points on both study days (See Fig 1). The anatomical image was recorded at T_-15min_. BOLD-signal was recorded at baseline (T_-5_), after infusion of CGRP or Placebo (T_40_) and 15 min after sumatriptan injection (T_60_). Subcutaneous injection of sumatriptan has a T_1/2_ of 2h with a maximum serum concentration 10 min after injection and a bioavailability of 96% [19, 20].

#### Headache

Headache intensity was recorded at 0, 5, 10, 15, 25, 35, 45, 55, 65 min on a verbal rating scale (VRS) from 0 to 10 [0, no headache; 1, a very mild headache (including a feeling of pressing or throbbing); 10, worst imaginable headache] [21].

#### Evoked Pain

**Fig 2:**
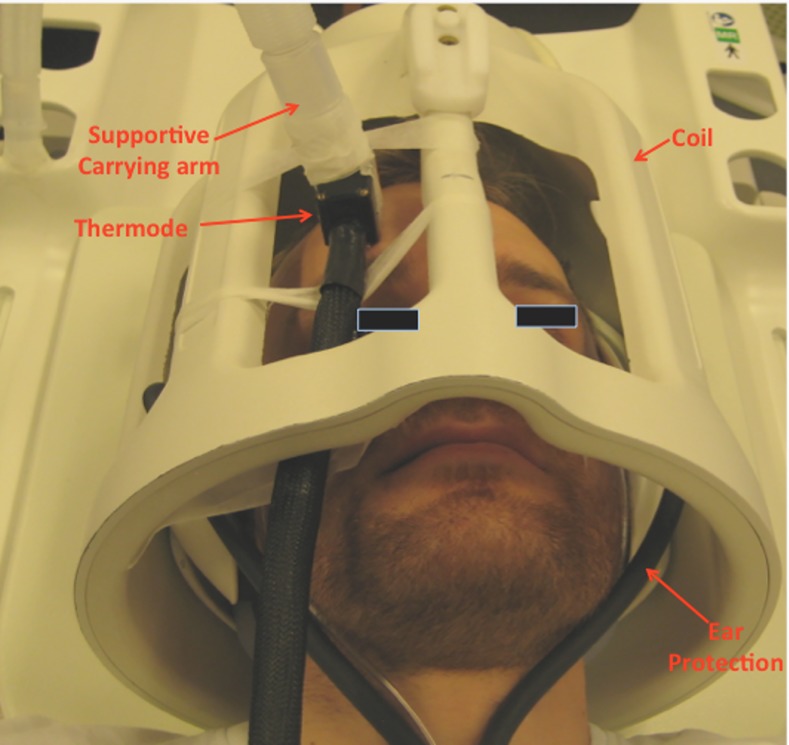
Study Setup: Reconstruction of the study setup. The subjects would be placed in the supine position inside the MRI scanner. The upper boarder of the eyebrows was used to centralize the subjects position in the scanner. The thermode was fitted in between the bars of the coil and then attached to the V1 area of the forehead. A custom build MRI compatible carrying arm and adhesive tape was used to ensure that only the heating element of the thermode came in contact with the skin. The subjects would be wearing ear protection under the whole duration of the MRI scans.

Noxious heat stimuli were delivered to the dominant side of the forehead (V1 branch of the trigeminal nerve) on both study days using a MRI compatible 1.6 x 1.6 cm contact Thermode (TSA-II with filter, Medoc Advanced Medical Systems, Ramat Yishai, Israel). The thermode permits a temporal and temperature controlled heat pain stimulation. The temperature applied for the pain stimulation was determined individually for each subject before the start of the study, as the temperature that resulted in a pain response of 5 on the Numerical Rating Scale (NRS) [0, no pain; 10, Worst imaginable pain][22]. This pre-defined temperature was then applied throughout both study days (For study setup see Fig 2).

**Fig 3:**
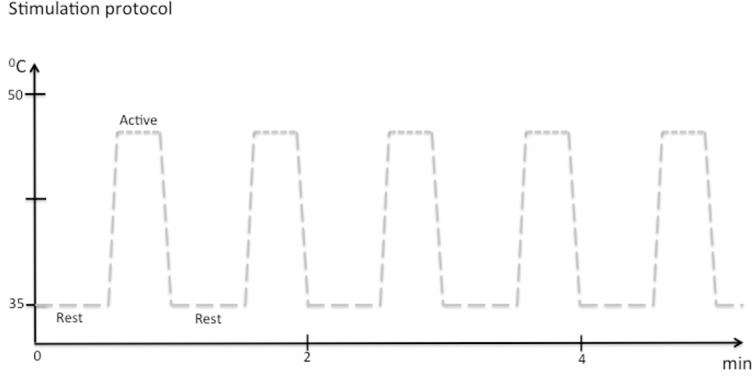
Stimulation paradigm: During functional imaging, blocks of noxious heat stimulation (pain) were delivered from baseline blocks (no pain) at 35°C. For noxious heat we chose the pre-determined individual temperature that corresponded to 5 on the numeric rating scale (NRS). One scan session thus consisted of 6 baseline blocks (30 s/block) that was interleaved by 5 noxious heat blocks (25 s/blocks), not including the ramp periods. The rate of temperature change was 4°C/s. The ramps were modeled in defining the explanatory variables (EVs) for fMRI data analysis.

During functional imaging, blocks of noxious heat stimulation (pain) with the pre-determined temperature were delivered from baseline blocks (no pain) at 35°C (See Fig 3).

### Data acquisition and Imaging protocols

*Anatomical Images*: Anatomical images were acquired using a T1-weighted 3D turbo field echo sequence (128 sagittal slices 1.2 mm thick; in-plane resolution 2.4 x 2 mm: repetition time 9.7 s; echo time 4.6 ms; flip angle 8, scan duration of 491 s).

*BOLD-signal*: BOLD functional imaging utilized a gradient echo EPI sequence (39 slices 3.0 mm thick; slice gap 0.06 mm; field of view 192 x 192 mm; in-plane acquired resolution 3 x 3 mm; repetition time 3.0 s; echo time 35 ms, flip angle 90°. 96 volumes per 4 min 48 sec scan session.

Slices were oriented coronally, parallel to the posterior border of the medulla oblongata, covering all structures between the brain cortex and the brainstem. The brainstem was centered in the middle of the functional scans.

### fMRI Analysis

Functional images were analyzed using FMRIB Software Library (FSL) (www.fmrib.ox.ac.uk/fsl). The first two volumes of each functional scan were removed to allow equilibration of image intensities. The images from subjects who had received stimulation on the left side (8 subjects) of their face were flipped along the sagittal axis (left/right) so they could be compared to those who received stimulation to the right side of their face (19 subjects). Additional pre-processing steps included, motion-correction, brain extraction, spatial smoothing (5 mm) and temporal filtering (high pass 100 s).

Whole brain analysis was performed on the data. A full quality assurance was performed prior to the statistical analysis. All scans that passed quality assurance parameters for motion correction (< 3 mm), registration to a standard space, and visual inspection of brain extraction and were included in the following statistical analysis. Statistical results were registered to a standard atlas (MNI-152 atlas). Results were visually inspected and it was individually ensured that there was a full set of scans during noxious heat stimulation (baseline, during CGRP/Placebo infusion and after sumatriptan injection).

Individual generalized linear model (GLM) results were then fed into a fixed effects analysis model using FLAME (FMRIB's Local Analysis of Mixed Effects). The output of this was checked for outliers using regression diagnostics.

Comparisons were made between the following BOLD scans for each study day: i. T_40_ (during infusion) vs. T_-5_ (baseline); ii. T_60_ (after sumatriptan injection) vs T_-5_ (baseline), and iii. T_60_ (after sumatriptan injection) vs. T_40_ (during infusion/pre-sumatriptan). Thresholds for the comparisons were determined using a mixture modeling approach [23]. The mixture modeling approach assigns a posterior probability to each voxel according to a classification of active, de-active or null class [23]. Based on that classification, we assign a voxel as active if it has more then 50% change of belonging (posterior probability > 0.5) to the class activation. Based on the thresholding we performed an analysis of clusters of activation to determine localized activity and its extent.

### Statistical Analysis

Headache- and pain scores are presented as median and range. All remaining values are presented as mean ± SD. The area under the response curve (AUC) for headache score was calculated according to the trapezium rule.

The primary endpoints were differences in BOLD-signal: i) at T_40_ (during infusion) compared to T_-5_ (baseline); ii) at T_60_ (after sumatriptan injection) compared to T_-5_ (baseline); iii) at T_60_ (after sumatriptan injection) compared to T_40_ (during infusion/pre-sumatriptan) on the CGRP day and placebo respectively; iv) BOLD-signal at baseline on the placebo day compared to baseline on the CGRP day.

It was predetermined that direct comparison between the CGRP and placebo scans would only be performed if there were either differences in baseline BOLD-signal between the two study days or differences in BOLD-signal at T_-5_ (baseline) and T_40_ on the placebo day.

In addition, we tested for difference in AUC for headache score in the period 0 to 45 min between the two experimental days. We also tested for differences in headache score after sumatriptan on both experimental days.

Differences in physiological variables were tested using two-way ANOVA. Headache- and pain scores were tested using Wilcoxon signed rank test. Regarding the remaining non-imaging data we tested the difference between two experimental conditions using a paired, two-way Students *t* test.

Five percent (*p < 0*.*05*) was accepted as the level of significance. All analysis was performed using SPSS for Mac 16.0 (SPSS Inc., Chicago, IL). For imaging data group contrast statistical maps were analyzed with a modified false-discovery rate (FDR) method [23] that uses mixture model to determine the null component. FDR is a statistical method used to correct for multiple comparisons and is the method preferred when performing BOLD-fMRI analysis by FSL [23, 24]. Thresholds of activation/deactivation were then used to determine clusters of activation (peak and volume) using in-house matlab programs (Mathworks Inc Natick, MA USA).

## Results

### Subjects

Of the 31 participants enrolled, 27 completed the study (10 M, 17F, Ratio: 1:1.7), with a mean age of 24.7 years (range 19 to 37 years). Two participants were excluded due to claustrophobia. One participant did not report for the second study day and we lost contact. One participant did not complete the second study day due to technical problems with thermode. No functional scan was removed due to excessive movement or movement artifacts.

### Physiological Measures

Baseline blood samples showed normal hematocrit, potassium- and sodium levels. Blood pressure, heart rate, oxygen saturation, end-tidal PCO_2_ are shown in Table 1. No statistical significant differences were recorded between time points within each study day or between study days (*P > 0*.*05*).

**Table 1:** Physiological data. Blood pressure, heart rate and End Tidal CO_2_ in 27 healthy volunteers. No statistical significant differences were recorded between time points within each study day or between study days (*P > 0*.*05*).

	**CGRP Day**							
	**Blood Pressure**							
**Time**	**Systolic (**±**SD)**		**Diastolic (**±**SD)**		**Heart rate (**±**SD)**		**End Tidal CO2**	
(min)	(mm Hg)		(mm Hg)		(/min)		(mm Hg)	
Baseline	109.9	(±23.5)	66.9	(±8.4)	63.3	(±11.3)	5.0	(±0.58)
T5	114.6	(±8.6)	64.7	(±6.7)	64.4	(±10.4)	5.1	(±0.56)
T10	114.6	(±10.2)	65.0	(±8.2)	65.0	(±11.1)	5.1	(±0.53)
T15	114.3	(±11.3)	63.1	(±7.4)	68.7	(±10.2)	5.0	(±0.57)
T25	115.5	(±10.4)	64.9	(±9.5)	71.8	(±12.0)	5.0	(±0.53)
T35	116.5	(±11.0)	64.5	(±8.6)	68.8	(±14.7)	5.1	(±0.46)
T45	116.7	(±10.3)	63.2	(±8.5)	66.0	(±13.5)	5.0	(±0.53)
T55	119.5	(±24.2)	76.3	(±8.6)	66.9	(±0.6)	5.0	(±0.49)
T65	121.8	(±9.8)	74.9	(±6.8)	64.6	(±11.0)	4.9	(±0.59)
	**Placebo Day**							
	**Blood Pressure**							
**Time**	**Systolic (**±**SD)**		**Diastolic (**±**SD)**		**Heart rate (**±**SD)**		**End Tidal CO2**	
(min)	(mm Hg)		(mm Hg)		(/min)		(mm Hg)	
Baseline	116.9	(±11.4)	67.3	(±8.3)	64.9	(±11.8)	5.1	(±0.48)
T5	117.0	(±11.7)	66.7	(±7.9)	63.6	(±11.9)	5.2	(±0.50)
T10	117.6	(±11.3)	67.7	(±8.3)	65.4	(±10.9)	5.2	(±0.47)
T15	116.9	(±11.5)	67.7	(±8.2)	65.5	(±10.9)	5.2	(±0.50)
T25	118.0	(±11.9)	67.4	(±7.6)	66.3	(±12.3)	5.1	(±0.51)
T35	117.9	(±9.6)	68.1	(±6.6)	67.1	(±11.8)	5.1	(±0.55)
T45	118.4	(±9.8)	69.5	(±9.5)	64.2	(±10.8)	5.0	(±0.52)
T55	127.1	(±11.2)	80.5	(±7.3)	67.7	(±9.7)	5.1	(±0.54)
T65	127.1	(±9.7)	77.7	(±8.3)	64.9	(±11.9)	5.1	(±0.51)

### Psychometric Measures

#### Headache

**Fig 4:**
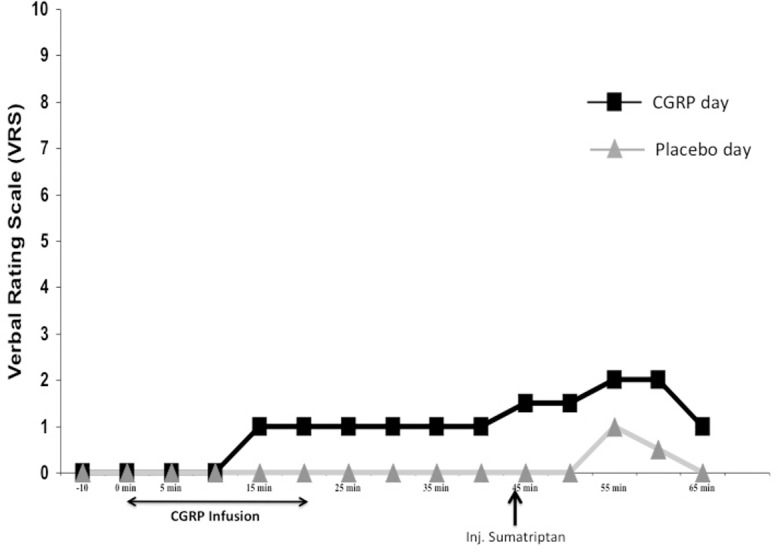
Headache score: Median headache score on the verbal rating scale (VRS) in 27 healthy volunteers. Black line with squares is the headache score on the CGRP day while gray line with triangles is headache score on the placebo day.

**Fig 5:**
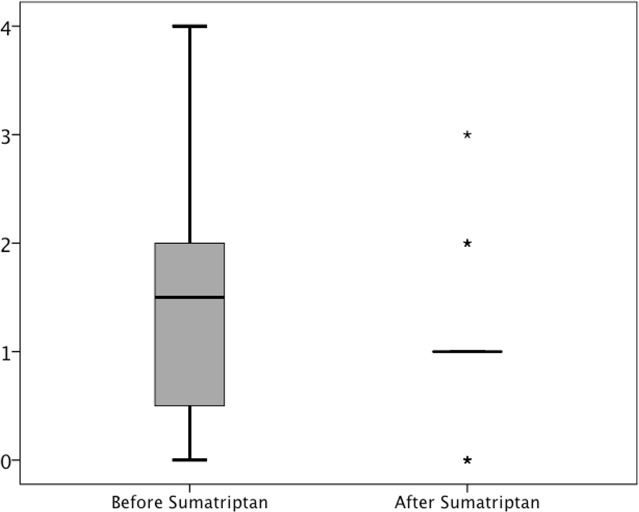
Headache score: Boxplot of median headache on the CGRP day before (T_40_) and after sumatriptan injection (T_65_). Stars (*) symbolize outliers. Before sumatriptan administration there was a median headache of 1.5 on the verbal rating scale (VRS) (range: 0–4). After treatment with sumatriptan the median headache intensity dropped to VRS 1.0 (range 0–3) *(P = 0*.*099)*.

21 (~78%) out of 27 participants reported immediate headache during CGRP infusion compared to seven (~26%) participants who reported headache during placebo (*P < 0*.*001*). The AUC for headache score (0–45 min) was larger on the CGRP day (median 70.0; Range 0–155), than on the placebo day (median 25.0; Range 0–95), (*P = 0*.*001*) (Fig 4). On the CGRP day sumatriptan did not significantly reduce median headache score although a tendency was recorded *(P = 0*.*099)* (See Fig 5).

#### Evoked Pain: Pain scores after heat stimulus

**Fig 6:**
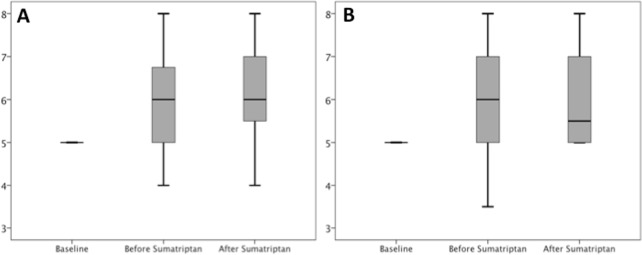
Pain score: Boxplot of pain scores (NRS) after noxious heat stimulation at baseline, before sumatriptan and after sumatriptan administration. A (left side) show the pain scores on the CGRP days. B (Right side) show the pain scores on the placebo day. There was no difference in pain score between the two experimental days at baseline (*P = 1*.*0*), before sumatriptan (*P = 0*.*87*) and after sumatriptan (*P = 0*.*36*) administration.

A mean temperature of 47.2°C ± 0.56 (Range 46°C–48°C) resulted in a pain score of 5 on the NRS scale at baseline. There was no difference in baseline NRS score between the two experimental days (*P = 1*.*0*). Median pain score increased from NRS 5 (pre-treatment) to NRS 6 (post-treatment) on both study days, and there were no differences between study days *(Z = -0*.*095*, *P = 0*.*92)*. Sumatriptan injection did not change the pain score on either experimental day (CGRP day; *Z = -1*.*44 P = 0*.*15*, *Placebo day; Z -0*.*062 P = 0*.*951*) (See Fig 6).

### Functional Imaging Results

#### Baseline

Table 2 shows the z-stats scores and coordinates for neuronal activation at baseline (before start of infusion). There was no difference in activation at baseline between the two study days.

**Table 2:** Contrast analysis results for painful heat functional MRI activation at baseline (before infusion of CGRP). NOTE: Two ROIs usually active in pain (anterior cingulate cortex and anterior insula) are not identified due to the distortion of the images.

Brain Region		Lat.	z-stat	X (mm)	Y (mm)	Z (mm)	Vol (cm^3^)
**Positive Activation**							
***Cortical***							
	*Frontal*						
	Middle	L	02.43	-34	10	42	00.22
	Supp Motor Area	L	02.97	-12	8	50	00.41
*P*	*Parietal*						
	SupraMarginal	R	02.31	66	-18	24	00.35
	Postcentral	L	02.05	-56	-22	26	00.28
	SupraMarginal	R	02.31	46	-32	38	00.34
	Inferior	R	03.77	50	-44	52	12.03
	*Occipital*						
	RolandicOperculum	L	02.07	-50	2	6	00.38
		R	02.32	40	-18	22	00.29
	*Temporal*						
	Middle	R	02.18	60	-30	-4	00.36
	Fusiform	L	03.16	-36	-74	-18	07.28
	*Insular*						
	InsulaPosterior	R	02.12	38	-10	22	00.25
	InsulaPosterior	L	02.84	-44	-10	4	0,057
***Brainstem / Cerebellum***							
	Pons	R	02.50	6	-18	-20	00.25
	Vermis 4 5		02.48	-2	-58	-18	00.58
	Cerebellum Crus1	R	03.03	40	-68	-30	01.03
		R	02.44	44	-78	-34	01.19
		R	02.23	26	-80	-24	00.31
	Cerebellum 6	R	02.22	30	-78	-20	00.25
	Cerebellum Crus2	R	02.37	36	-80	-42	00.30
**Negative Activation**							
***Cortical***							
	*Frontal*						
	Superior_Orbital	R	03.14	28	-12	66	00.28
	Precentral	R	02.65	40	-14	62	00.25
		R	03.01	28	-20	66	01.04
		R	02.08	22	-32	70	0.052
	*Parietal*						
	Precuneus	L	05.35	-2	-40	64	10.53
	*Temporal*						
	Fusiform	L	03.24	-40	-42	-24	00.38
	Lingual	L	03.62	-12	-46	2	09.13
	Middle	L	03.24	-40	-50	10	00.34
	*Cingulum*						
	Middle	L	02.53	-6	-40	42	00.29
	*Parahippocampus*						
	Parahippocampal	L	04.07	-24	4	-32	15.33
***Sub-Cortical***							
	Caudate	L	03.05	-10	20	4	0.088
		R	05.03	10	18	0	00.49
		L	02.89	-6	16	6	00.25
		R	05.26	10	14	0	00.06
		L	04.34	-6	6	-6	00.66
	Nac	R	04.45	6	6	-8	00.85
***Brainstem/Cerebellum***							
	Cerebellum 4 5	L	03.08	-26	-36	-26	00.27
	Cerebellum 8	R	03.13	24	-62	-56	00.51

#### CGRP infusion

**Fig 7:**
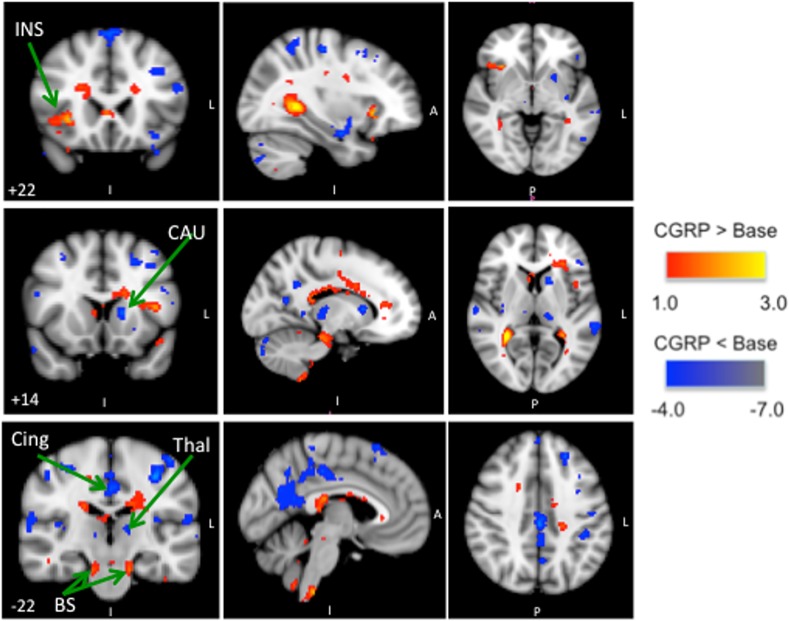
BOLD-signal during CGRP infusion: Group BOLD-fMRI results for noxious heat simulations to the V1 area on the CGRP day; differences between neuronal activation at T_40_ (after CGRP infusion) and T_-5_ (baseline). The activated regions-of-interest are shown in three projections. CGRP infusion resulted in positive activation in insula and bilateral activation in the brainstem, while negative activation was recorded in the caudate nuclei, thalamus and cingulate cortex.

At 40 min after of start of CGRP infusion we recorded bilateral significantly increased BOLD-signal in the brainstem and unilateral increased BOLD-signal in the insula while decreased BOLD-signal was recorded in the caudate nuclei, thalamus and cingulate cortex (see Fig 7, Table 3).

**Table 3:** Contrast analysis results for painful heat functional MRI activation after CGRP infusion.

Brain Region	Lat.	z-stat	X (mm)		Y (mm)	Z (mm)	Vol (cm^3^)
**Positive Activation**							
***Cortical***							
*Frontal*							
Inferior Operculum	L	02.80		-40	12	12	1.52
Inferior Triangular	L	02.45		-30	32	4	0.78
Inferior Orbital	R	01.27		40	22	-2	1.18
*Insular*							
InsulaAnterior	R	03.19		32	22	-2	00.67
	L	01.90		-32	0	16	00.66
***Brainstem / Cerebellum***							
Brain Stem / Pons	R	02.61		14	-20	-24	00.58
	L	02.28		-14	-22	-24	00.58
**Negative Activation**							
***Cortical***							
*Frontal*							
Supp Motor Area	R	-05.71		6	18	64	01.70
Precentral Lobule	L	-05.05		-8	-38	62	00.83
Precentral	L	-05.24		-30	-8	62	01.14
	R	-05.24		32	-32	54	01.73
	L	-05.68		-52	0	46	00.85
Middle	L	-04.65		-30	0	50	02.24
	L	-05.06		-24	12	44	00.78
	L	-04.70		-36	16	42	00.78
	L	-05.30		-24	36	40	01.70
	L	-04.93		-40	24	34	00.86
	L	-04.85		-28	34	32	00.82
Inferior Triangular	L	-05.06		-48	24	22	01.78
	L	-04.65		-46	32	8	01.16
Inferior Operculum	R	-04.56		48	6	18	00.70
Inferior Orbital	L	-04.84		-36	24	-16	00.73
*Parietal*							
Superior	R	-05.00		34	-46	60	00.86
Inferior	R	-04.80		30	-50	52	01.08
	L	-05.01		-46	-36	50	01.31
	L	-04.96		-40	-36	40	01.23
Postcentral	R	-05.91		42	-36	56	01.24
	R	-04.99		38	-34	50	00.86
	L	-05.48		-34	-34	62	01.22
	L	-04.85		-42	-42	58	01.05
	L	-04.72		-24	-46	56	00.70
	L	-05.61		-54	-12	50	01.74
	L	-06.06		-34	-24	48	02.35
	L	-04.65		-58	-14	16	00.82
Supramarginal	L	-04.82		-60	-24	16	00.96
Precuneus	L	-04.56		-4	-46	38	01.50
	L	-05.02		-6	-54	18	01.06
Angular	L	-05.46		-52	-52	34	02.02
	L	-05.02		-6	-54	18	01.06
*Occipital*							
Cuneus	L	-04.89		-4	-66	26	02.03
Calcarine	R	-05.06		10	-70	18	00.89
	R	-04.54		10	-60	18	02.22
	R	-04.75		12	-66	10	00.85
	L	-05.19		-12	-62	14	03.63
*Cingulum*							
Middle	L	-05.11		0	-40	50	03.21
	L	-04.67		-2	-40	42	00.86
	L	-05.61		0	-26	40	03.99
	L	-04.54		0	28	34	00.70
Posterior	L	-05.04		0	-48	24	01.42
***Sub-Cortical***							
Caudate	L	-05.17		-14	14	6	00.90
Thalamus	L	-04.80		-14	-26	6	00.90
Putamen	L	-04.85		-24	10	-8	01.09
Amygdala	R	-05.02		28	2	-12	01.10
Hippocampus	R	-05.14		32	-6	20	01.10
***Brainstem/Cerebellum***							
Cerebellum Crus 1	R	-04.74		30	-80	-44	02.95
Cerebellum Crus 2	L	-04.64		-14	-82	-24	01.10
Cerebellum 8	R	-04.67		10	-66	-46	00.85

**Fig 8:**
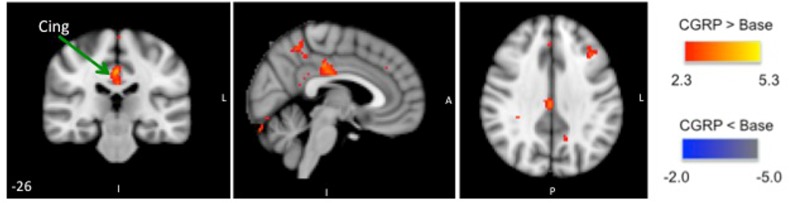
BOLD-signal after sumatriptan administration: Group BOLD-fMRI results for noxious heat simulations to the V1 area on the CGRP day; differences between neuronal activation at T_60_ (after sumatriptan) and T_40_ (during CGRP infusion/before sumatriptan). The activated regions-of-interest are shown in three projections. Sumatriptan injection resulted in positive activation of the cingulate cortex.

Injection of sumatriptan reversed CGRP induced changes compared to baseline (T_-5_). Comparison between the BOLD scan before and after sumatriptan (T_40_ compared to T_60_) revealed increased BOLD-signal in the cingulate cortex (See Fig 8, Table 4).

**Table 4:** Contrast analysis results for painful heat functional MRI activation after sumatriptan administration on the CGRP day.

Brain Region	Lat.	z-stat	X (mm)		Y (mm)	Z (mm)	Vol (cm^3^)
**Positive Activation**							
***Cortical***							
*Frontal*							
Supp Motor Area	L	04.23		-4	6	44	00.33
**Negative Activation**							
***Cortical***							
*Frontal*							
Middle	L	-2.81		-38	34	44	00.27
	L	-3.11		-22	26	48	01.53
Inf. Triangular	L	-3.63		-52	22	24	00.38
	L	-3.59		-52	22	10	0.042
*Parietal*							
Postcentral	L	-3.31		-50	-18	36	00.38
Postcentral	L	-3.33		-54	-26	54	00.46
Angular	L	-3.33		-42	-58	24	00.33
*Insular*							
InsulaAnterior	L	-3.35		-38	18	-6	00.26
***Sub-Cortical***							
Thalamus	L	-3.11		-6	-14	14	00.24
***Brainstem/Cerebellum***							
Cerebellum Crus 2	R	-3.31		42	-74	-40	00.43

#### Placebo infusion

At 40 min after start of placebo infusion we recorded no statistically significant changes in BOLD-signal compared to baseline.

**Fig 9:**
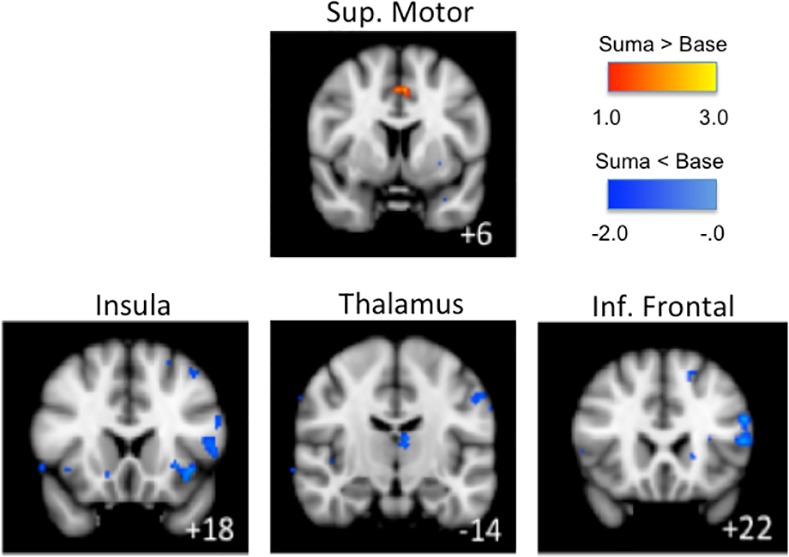
BOLD-signal after sumatriptan administration: Group BOLD-fMRI results for noxious stimulation to the V1 area on the placebo day. Here differences in neuronal activation between the BOLD-fMRI scan at T_-5_ (baseline) and T_60_ (after sumatriptan) is shown. Sumatriptan injection resulted in positive activation of the supplementary motor area (sup. motor area) and negative activation in the inferior frontal cortex (inf. frontal), insula and thalamus.

Injection of sumatriptan induced significantly increased BOLD-signal in the supplementary motor area and decreased BOLD-signal in the inferior frontal cortex, post-central cortex, anterior insula, thalamus and cerebellum compared to both baseline (T_-5_) and to the pre-sumatriptan (T_40_) measurements_._ (See Fig 9).

## Discussion

The major findings of the present study were that intravenous infusion of CGRP modulated BOLD-signal in the brain in response to noxious heat stimuli of the trigeminal nerve without changing pain scores. Furthermore, the anti-migraine drug sumatriptan reversed these changes.

### Systemic CGRP: central or peripheral effect?

We found that systemic administration of CGRP was associated with altered functional activation in the CNS as recorded by BOLD-fMRI. CGRP is widely distributed in the trigeminal pain pathway at the peripheral and central levels [2, 25–29]. Mechanisms responsible for CGRP induced head pain or migraine are complex and the question is whether intravenous CGRP: 1) crosses the blood brain barrier (BBB) and modulates nociception in the CNS; 2) activates and sensitizes trigeminal sensory afferents. Intravenous infusion of CGRP does not affect hemodynamics (blood pressure, heart rate, end-tidal CO_2_) [10, 14, 18], the cerebral blood flow (CBF) [13, 25] and visual processing [18] in man. Injection of CGRP directly into human skin or temporal muscle (i.e. in the areas of cutaneous and muscle distribution of the trigeminal afferents) did *not* elicit pain [30]. Furthermore, CGRP does not activate or sensitize meningeal nociceptors in rodents [31], nor does intra-thecal CGRP administration alone yield any changes [32]. In addition, CGRP reduces the discharge frequency of the wide dynamic range neurons in rats following electrical stimulation of the hind paw [33, 34], and modulates background activity and responses to brush, press and pinch to the skin of the hind paw [35, 36]. Pre-treatment with CGRP receptor-antagonists reduce capsaisin-evoked sensitization, while post-treatment with CGRP dose-dependently restores capsaisin-evoked sensitization in rats [37]. Collectively, these data suggests that CGRP exerts its action outside of the BBB and modulates sensory processing, without direct activating or sensitizing effects.

### Altered BOLD-signals in the brain after CGRP infusion

In the present study we examined BOLD-signal after noxious stimulation of ophthalmic (V1) branch of the trigeminal nerve before and after intravenous infusion of CGRP. We used noxious heat stimulation, which is a robust well-validated method that has been applied in several pain fMRI-studies [16, 38–40]. Stimulation of the ophthalmic nerve was chosen because of the trigeminal pain pathways importance in the pathophysiology of headaches.

At baseline (before infusion), heat stimulation to the forehead resulted in significant somatotropic activation of pain-related brain centers similar to previous studies [41–44]. We observed a dropout artifact in relation to the thermode. Dropout artifacts and distortions are commonly observed when thermodes are applied to the forehead, close to the brain. To limit the effects of these distortions the slices were oriented coronally during BOLD-fMRI scans and a filter was applied to the thermode. We were not able to visualize expected anterior cingulate cortex (ACC) activation as the area was covered by the thermode (see Table 2 for z-stat scores at baseline).

After CGRP infusion we recorded changes in brain areas related to ascending pain-pathways. Specifically we recorded increased BOLD-signal in the brainstem and insula and decreased BOLD-signal in the caudate nuclei, thalamus and cingulate cortex. Brainstem activation is reported during head pain conditions such as tooth pain [44], primary headaches [45–47] and in other pain conditions such as irritable bowel syndrome [48–50], fibromyalgia [51], angina pectoris [52] and osteoarthritis [53]. The question is whether the recorded changes are due to CGRP induced headache or due to modulation of neuronal pain processing in response to noxious heat stimuli? CGRP is widely distributed in trigeminal nuclei in the brainstem [5, 54–59]. CGRP induced very mild headache (median headache score = 1.5 [which correspond to pre-pain]). Furthermore the self reported pain scores in response to noxious heat stimuli after CGRP remained unchanged. In a previous study only increased headache score after high doses of glyceryl trinitrate (GTN) was associated with changes in mechanical pain thresholds [60]. While a study of allodynia reported a low consistency between pain-thresholds in different stimulation modalities [61]. The lack of change in self-reported pain scores could therefore both be explained as a dose-response relationship due to low headache score and because of the pain stimulation modality in question.

This could suggest that the increased BOLD-signal in the brainstem is due to modulation of nociceptive input by CGRP. We recorded bilateral decreased activation in thalamus after CGRP infusion. The thalamus plays an important role in acute pain and in development of sensitivity to pain [43]. Decreased thalamic activation has been associated with modulation of pain [62] and is furthermore reported in subjects that are highly sensitive to noxious heat pain [63] or in patients with chronic neuropathic pain [62]. Our finding of decreased thalamic activation is puzzling and could indicate that CGRP may induce increased sensitivity to pain leading to reduced inhibition, increased signal transduction or reduced localization and discrimination of stimuli [64]. With the present imaging resolution we could not localize the activation to specific nuclei since it was not possible to distinguish between the respective nuclei with certainty. At baseline noxious heat stimulation resulted in bilateral insula activation. Insula activation is correlated with the intensity of pain stimulation [65, 66] resulting in bilateral insula activation after noxious heat stimulation [63, 65, 67]. Interestingly, following CGRP infusion only unilateral increased activation of insula was recorded while none of the participants reported unilateral headaches. Unilateral activation of insula has previously been recorded in pain studies of migraine attacks [45, 68]. Insula is extensively connected both to pre-frontal cortex and ACC [69]. Co-activation of insula and ACC have previously been observed [70]. However, the issue is more complex. Brain regions including the ACC and insula have multiple functions but also share some unusual anatomical features such as von Economo neurons [71]. For example the insula is involved in pain, autonomic function, interoception, salience, and awareness including detection error [72–74]. Similarly the ACC is also involved in functions that include emotion, descending analgesia, empathy, attention and salience [75, 76]. Thus, while anatomical data shows connections between the insula and numerous brain areas including anterior cingulate [77], specificially the anterior-middle region of the insula and the dorsal anterior cingulate [78], the functional relationship including co-activation [79, 80] still remains unclear [81] but clearly can be postulated that different responses (activations) may take place in these structures [78] as a result of an independent functional (e.g., the insula may be more ‘sensitive’ to salience processing) processes. The role of pharmacological agents in altering the interplay between these systems (whether peripherally or centrally acting) remains unknown.

Increased activation in caudate nuclei is associated with pain inhibition [82, 83]. Activity in the caudate nucleus during pain may also correlate to sensory activity [83]. But if this were the case a decreased activity would indicate decreased sensory integration, which should have resulted in concomitant decreased activity in the somatosensory cortex and insula [69, 84]. Since this did not occur it is most likely that the caudate nucleus activation is instead associated with pain inhibition. There is generally consensus that the cingulate cortex plays a pivotal region for emotions and for avoidance behavior during pain [85]. The middle cingulate cortex (MCC) have motor areas that project to both the spinal cord and motor cortex and is involved in response selection [86]. Decreased activity in MCC in response to heat nociception after CGRP may reflect functionally impaired or decreased stimulus localization without altering the pain affect *per see* [87].

Taken together the recorded functional neuronal changes suggest nociceptive modulation from the peripheral nerve system (PNS) in response to noxious heat.

### Sumatriptan induced changes in BOLD-signal

In the present study the sumatriptan intervention was performed to elucidate possible modulation of central pathways after CGRP infusion. We administered sumatriptan subcutaneously 45 min after intravenous administration of CGRP or placebo. BOLD-signal was then recorded 15 min after sumatriptan injection by application of pain stimulation (noxious heat) to the forehead (V1 area of the trigeminal nerve).

We found that following sumatriptan administration the subjects experienced a transient worsening of the headache. This is a well known side-effect to sumatriptan [88]. On the CGRP day sumatriptan reduced the headache with 0.5 on the VRS scale while amelioration of headache was not achieved. This is probably because the healthy subjects only experienced mild headache (VRS = 1.5) and because they were only observed for at very short time-period following sumatriptan administration (15 min). Sumatriptan also reversed the CGRP induced functional changes in response to heat noxious stimuli. Further analysis showed that compared to the CGRP-induced functional changes, sumatriptan administration resulted in additional activation of the cingulate cortex.

Even though sumatriptan is a potent vasoconstrictor, studies of healthy subjects [10, 89] and migraine patients [14, 90] have indicated that sumatriptan does not cross the blood-brain-barrier (BBB) to a large extend and that its anti-nociceptive mode-of-action is unlikely vasoconstriction. Instead it is suggested that sumatriptan inhibits neuronal transmission from first to second order neurons at the trigeminospinal level [31].

Only two previous studies have examined neuronal activation by MRI after sumatriptan administration. Krämer et al. found that after peripheral stimulation (brush), sumatriptan administration resulted in decreased activation of posterior insula while increased activation was recorded in anterior insula, orbito frontal cortex, medial thalamus and ACC [91]. A resent fMRI study in normal volunteers reported that following electrical stimulation to the leg sumatriptan administration resulted in increased activation of secondary somatosentory cortex (SII), Insula, medial thalamus and ACC [92]. Interestingly, here participants also reported increased head pain after sumatriptan. Sumatriptan is primarily used for treatment of headaches. It is therefore remarkable that none of the previous studies have examined the effects of sumatriptan on headpain (i.e. nociceptive activation of the trigeminal pain pathway and/or experimental/spontaneous headache). Therefore the previous studies only offer limited insights into interpretation of the present results.

With respect to the limitations of this study, and given that neither CGRP [10, 12, 13] nor sumatriptan [10, 89, 93] crosses the BBB or affects the BOLD-signal *per see* [18] our data suggest that sumatriptan reverses changes in BOLD-signals induced by CGRP by modulation of sensory afferent transmission to the CNS. This may indirectly affect the cingulate cortex, which may in turn play an important role in inhibition of head pain.

## Conclusion

We found that intravenous infusion of CGRP in healthy volunteers is associated with mild headache and increased neuronal activity predominantly in the brainstem and insula; decreased neuronal activity in the caudate nuclei, thalamus and cingulate cortex. Sumatriptan reversed these changes probably by inhibiting pain transmission from the periphery to the brain without amelioration of the headache. We suggest that systemic CGRP modulates nociceptive transmission in the trigeminal pain pathways in response to heat noxious stimuli.
